# The COVID-19 pandemic: impact on surgical departments of non-university hospitals

**DOI:** 10.1186/s12893-020-00970-x

**Published:** 2020-12-03

**Authors:** Christian Stöß, Marcella Steffani, Kay Kohlhaw, Claudia Rudroff, Ludger Staib, Daniel Hartmann, Helmut Friess, Michael W. Müller

**Affiliations:** 1 Department of Surgery, Klinikum rechts der Isar, Technical University of Munich, School of Medicine, Ismaninger St 22, 81675 Munich, Germany; 2 Clinic of General, Visceral, MIS and Vascular Surgery, Clinical Centre Borna, Borna, Germany; 3 Clinic of General and Visceral Surgery, Evangelisches Klinikum Köln Weyertal, Cologne, Germany; 4 Clinic of General and Visceral Surgery, Clinical Centre Esslingen, Esslingen, Germany; 5 Clinic of General and Visceral Surgery, Clinical Centre Ludwigsburg-Bietigheim, Ludwigsburg, Germany

**Keywords:** COVID-19 pandemic, Surgical care, Financial loss, Surgery, Non-university hospitals

## Abstract

**Background:**

During the first wave of the COVID-19 pandemic, German health care centres were restructured for the treatment of COVID-19 patients. This was accompanied by the suspension of the surgical programme. The aim of the survey was to determine the effects of COVID-19 on surgical care in non-university hospitals in Germany.

**Methods:**

This cross-sectional study was based on an anonymous online survey, which was accessible from April 24th to May 10th, 2020 for surgeons of the *Konvent der leitenden Krankenhauschirurgen (Convention of leading Hospital Surgeons)* in Germany. The analysis comprised of 22.8% (n = 148/649) completed surveys.

**Results:**

Communication and cooperation with authorities, hospital administration and other departments were largely considered sufficient. In the early phase of the COVID-19 pandemic, 28.4% (n = 42/148) of the respondents complained about a short supply of protective equipment available for the hospital staff. 7.4% (n = 11/148) of the participants stated that emergency operations had to be postponed or rescheduled. A decreased quantity of emergency surgical procedures and a decreased number of surgical emergency patients treated in the emergency room was reported in 43.9% (n = 65/148) and 63.5% (n = 94/148), respectively. Consultation and treatment of oncological patients in the outpatient clinic was decreased in 54.1% (n = 80/148) of the surveyed hospitals. To increase the capacity for COVID-19 patients, a reduction of bed and operating room occupancy of 50.8 ± 19.3% and 54.2 ± 19.1% were reported, respectively. Therefore, 90.5% (n = 134/148) of all participants expected a loss of revenue of 28.2 ± 12.9% in 2020.

**Conclusion:**

The first wave of the COVID-19 pandemic had a significant impact on surgical care in Germany. The reduction in the bed and the operating room capacity may have lead to considerable delays in urgent and semi-elective surgical interventions. In addition to the risk of worsening patient care, we anticipate severe financial damage to the clinics in 2020 and beyond. National and supranational planning is urgently needed to ensure the surgical care of patients during the ongoing COVID-19 pandemic.

## Background

At the end of December 2019, an outbreak of an interstitial lung disease caused by a novel type of coronavirus (SARS-CoV-2) was first reported in the city of Wuhan, China [1]. The World Health Organization (WHO) subsequently designated it as the Coronavirus Disease 2019 (COVID-19) [2, 3]. One month later, the COVID-19 outbreak was declared a pandemic [4]. The rapid global spread of the viral infections and disease led to the introduction of far-reaching containment and reduction strategies in the affected countries around the world. To provide hospital capacity, protective equipment and ventilators for an expected increasing number of COVID-19 patients, surgical disciplines in Germany were instructed to postpone all elective surgeries and to reallocate staff to the intensive care units and COVID-19 wards as needed [5]. Various national surgical societies and associations published statements on the guidance for triage and urgent surgical interventions that were still considered feasible or mandatory [6–8]. Many surgical units were massively affected by restructuring measures [9, 10]. At the end of April 2020, during this survey, the German Government determined that hospital capacities should gradually resume elective interventions [5]. The long-term effects of the suspension of the elective surgical programme on the non-academic surgical departments in Germany are currently not foreseeable.

The present cross-sectional study aims to evaluate the impact of the global COVID-19 pandemic and subsequent governmental directives on surgical departments of non-university hospitals in Germany after the first infectious wave. In the survey conducted, data were collected on experiences regarding the effect of the governmental restrictions, on restructuring and financial burdens for the surgical departments, as well as on the assessment of future developments. The results may be helpful for other European countries in adapting containment strategies or resuming elective surgeries in order to regain high quality surgical care under the given circumstances, especially since further waves of the pandemic are expected.

## Methods

### Study design

For the present cross-sectional study, the members of the *Konvent der leitenden Krankenhauschirurgen (Convention of leading Hospital Surgeons, KLK),* an association of chief surgeons in Germany, were invited to an anonymous online survey from April 24th to May 10th, 2020 via email [11]. Participation in the survey was voluntary and anonymous. 23.4% (n = 152/649) of all surgeons contacted took part in the survey. A total of 4 completed surveys had to be excluded from the analysis because they were answered by surgeons from university hospitals. Thus, 22.8% (n = 148/649) answers of non-university surgeons were analyzed. See Additional file 1: Table S1 for the membership structure of *KLK.*

### Survey

A commercial provider was used for conducting the online survey (Google Forms, https://docs.google.com/forms; Google Inc. Mountain View, CA, USA), which comprised of 67 individual questions and statements in 8 categories (general information, politics, health authorities, hospital administration, communication with other medical departments, consequences of restructuring, effects on case numbers, outlook for the time after the COVID-19 pandemic). A combination of a bipolar, numbered Likert scale (1 = "Strongly agree", 2 = "Agree", 3 = "Neutral", 4 = "Disagree" and 5 = "Strongly disagree"), closed (Yes/No/Unknown) and open questions was used.

In the first section, general characteristics (9 questions) were collected from the respondents. In the second (Politics, 5 questions) and third section (Health Authorities, 4 questions), respondents were asked to evaluate communication and actions taken by the federal and state governments and health authorities in relation to the COVID-19 pandemic. In the fourth section (Hospital administration, 8 questions), the cooperation of the hospital administration was asked. The fifth category (Cooperation with other specialties during the COVID-19 pandemic, 5 questions) examined the cooperation with other specialist departments and in particular the continuation of the interdisciplinary tumour board. Special emphasis was placed on the sixth and seventh category (Pandemic-related restructurings and current case numbers, 28 questions), which assessed the preliminary effects of the COVID-19 pandemic caused by the suspension of all elective surgeries and the reallocation of surgical personnel for the care of COVID-19 patients. Finally, in the eighth category (Outlook for the period after the COVID-19 pandemic, 8 questions) the chief surgeons were asked to evaluate possible future changes caused by the pandemic. The utilized questionnaire had previously been applied to investigate the effects of the COVID-19 pandemic on university hospitals in Germany [12]. The complete survey is provided in Additional file 2: Survey.

### Statistical analysis

Descriptive data analysis was performed with Microsoft Excel 2019 (Microsoft, Redmond, USA). The data are given as absolute and relative frequencies. For continuous variables, the mean value and the single standard deviation were calculated.

## Results

### General characteristics

The participants were mostly male (n = 139/148, 93.9%). The majority of the respondents were older than 50 years of age (83.8%, n = 124/148). 96.6% (n = 143/148) participants were head of their departments, the remaining 3.4% comprises of senior attendings. 7.4% (n = 11/148) of the respondents work at hospitals of maximum care (comprises of several specialties aside of a surgical/internal medicine department with highly differentiated medical-technical facilities, responsible for teaching and research), 35.8% (n = 53/148) work at specialized hospitals (comprises of several specialties aside of a surgical/internal medicine department) and 56.1% (n = 83/148) at general hospitals (comprises of at least one surgical and/or internal medicine department). With regard to hospital size (number of beds), more than 50% of the respondents work in hospitals with a 201–500 bed capacity. Most of the respondents (58.1%, n = 86/148) had 10–20 intensive care beds available in their respective hospital. 83 out of 148 participants (56.1%) reported that they perform less than 2,000 operations per year, 44/148 (29.7%) responded a case load of 2,000–3,000 operations and 15/148 (10.1%) reported more than 3,000 operations per year. Six participants did not specify the surgical case load (n = 6/148; 4.1%). See Table 1 for general characteristics of the study cohort.

**Table 1:** General characteristics

Variable	n (%)
*Age (in years)*	
< 30	0 (0)
31–50	23 (15.5)
> 50	124 (83.8)
Not specified	1 (0.7)
*Sex*	
Male	139 (93.9)
Female	6 (4.1)
Non-binary	1 (0.7)
Not specified	2 (1.4)
Profession*	
Head of department	143 (96.6)
Senior consultant	5 (3.4)
*Type of hospital*	
Maximum care hospital^1^	11 (7.4)
Specialized hospital^2^	53 (35.8)
General hospital^3^	83 (56.1)
Not specified	1 (0.7)
*Size of hospital (number of beds)*	
< 100	2 (1.4)
100–200	20 (13.5)
201–500	85 (57.4)
501–1000	34 (23.0)
> 1000	6 (4.1)
Not specified	1 (0.7)
*Intensive care capacity (number of intensive care beds)*	
< 10	23 (15.5)
10–20	86 (58.1)
21–50	29 (19.6)
51–100	8 (5.4)
> 100	2 (1.4)
Not specified	0 (0)
*Number of yearly performed operations*	
< 1000	7 (4.7)
1000–2000	76 (51.4)
2001–3000	44 (29.7)
> 3000	15 (10.1)
Not specified	6 (4.1)

^*^Answer was obligatory to fill in

^1^A hospital of maximum care comprises of several specialties aside of a surgical/internal medicine department with highly differentiated medical-technical facilities, responsible for teaching and research

^2^A specialized hospital comprises of several specialties aside of a surgical/internal medicine department

^3^A general hospital comprises of at least one surgical and/or internal medicine department

### Policy perception and cooperation with health authorities

**Fig. 1:**
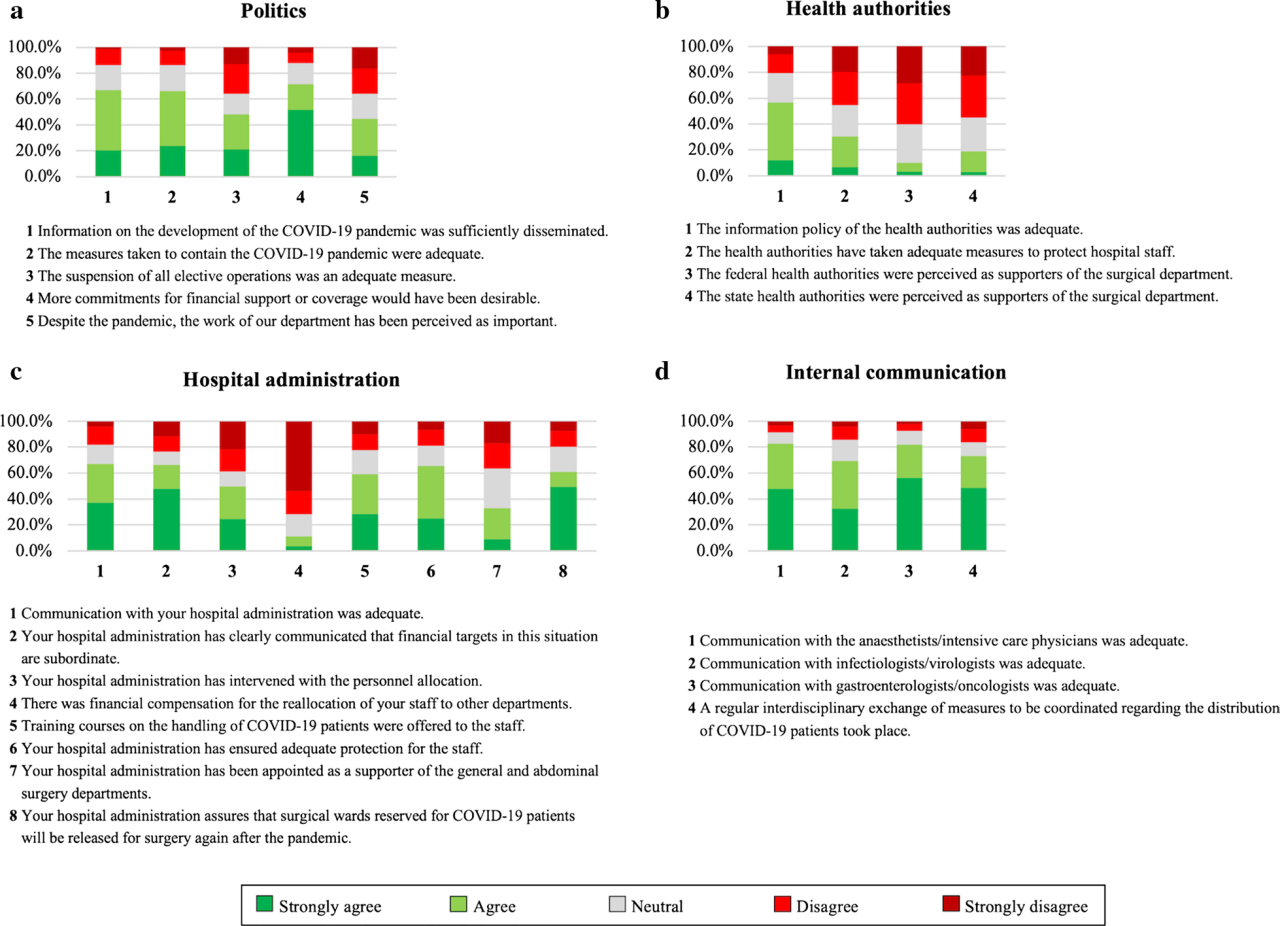
Results of questionnaire sections 1–4: **a** Politics, 5 questions **b** Health authorities, 4 questions **c** Hospital administration, 8 questions **d** Cooperation with other specialist departments, 5 questions

The statement that sufficient information about the COVID-19 pandemic was provided by the politics was fully supported by 20.3% (n = 30/148) and supported by 46.6% (n = 69/148) of the participants, while 12.2% (n = 18/148) disagreed or fully disagreed with this statement, 19.6% were neutral (n = 29/148) (Fig. 1a). The majority also agreed with the statement that the overall measures taken by politicians to contain the pandemic were adequate: 23.6% (n = 35/148) fully agreed and 42.6% (n = 63/148) agreed. In contrast, less than half of the participants fully supported or supported the suspension of the elective programme as an appropriate measure: 20.9% (n = 31/148) and 27.0% (n = 40/148), respectively. The majority of respondents indicated that they would have appreciated more financial support: A total of 51.7% (n = 76/147) fully agreed with the statement and 19.7% (n = 39/147) agreed with it.

The information policy of the health authorities was perceived as very positive by 12.2% (n = 18/148) and positive by 44.6% (n = 66/148). However, agreement on measures taken by health authorities to protect hospital staff was less: 6.8% (n = 10/148) fully agreed and 23.6% (n = 35/148) agreed. Support for surgical departments by both federal and state health authorities was perceived by the majority as low to very low (Fig. 1b).

### Internal communication with the hospital administration

48.0% (n = 71/148) of the participants strongly agreed and 18.2% (n = 27/148) agreed that their hospital management currently considers previously agreed financial targets to be of secondary importance (Fig. 1c); 23.7% (n = 25/148) did not agree with this statement. Financial compensation for the redeployment of staff to other departments was fully affirmed by 3.4% (n = 5/145) and 7.6% (n = 11/145) agreed; but overall 71.7% (n = 104/145) of respondents denied the statement. In 49.7% (n = 73/147) of the respondents, the hospital management intervened in the allocation of personnel; for a total of 38.8% (n = 57/147) this was not the case. Hospital administrations assured that the beds reserved for COVID-19 patients would be available for surgical patients again after the pandemic in 60.9% (fully agreed: n = 72/146; agreed: n = 17/146).

### Cooperation with other departments

Between 69.4–82.4% of the respondents (totally) agreed with the statements that the communication was adequate with the colleaques of the departments of anaesthesiology, intensive care, infectiology, virology, oncology and gastroenterology, respectively (Fig. 1d). One question addressed the interdisciplinary tumour board, which was carried out as usual in 16.2% (n = 24/148) of the hospitals. 39.9% (n = 59/148) stated that the tumour board was carried out with reduced staff and 27.0% (n = 40/148) corresponded via video conference. 11.5% (n = 17/148) reported that the tumour board was set out completely (5.4%; n = 8/148 not specified). See Additional file 3: Figure S1 for depiction of the answers regarding the interdisciplinary tumour board.

### Effects of the pandemic on departments for surgery

The survey showed that due to the COVID-19 pandemic, the bed capacity of German surgical departments was temporarily reduced by an average of 50.8 ± 19.3%. The occupancy rate of the reduced bed capacity was estimated at 49.6 ± 24.3%. The operating room capacity was reduced by an average of 54.2 ± 19.1%. The utilization of the reduced operating room capacity was 53.2 ± 27.9%. Outpatient clinic capacity was also reduced significantly with an average reduction of 69.3 ± 23.2% and the utilization was estimated to be 41.1 ± 30.8%. This is consistent with the finding that 97.3% (n = 144/148) of the respondents indicated that patients cancelled elective surgeries. According to study participants, other surgical departments were also affected by the reduction in bed and operating room capacity. 90.5% (n = 134/148) of all participants expected a loss of revenue in 2020 with an average of 28.2 ± 12.9%, while 6.1% (n = 9/148) expected no loss or even an increase in revenue (3.4%; n = 5/148 not specified). 45.3% (n = 67/148) of the study participants think that this will have financial consequences for their department (15.5%; n = 23/148 unknown); 30.4% (n = 45/148) agreed that it will have an impact on the personnel situation, as well (Table 2).

**Table 2:** Restructurings due to COVID-19 pandemic (Part 1)

Questions	Answer (in %) Mean ± single standard deviation
Estimate the current reduction in bed capacity in your department	50.8 (± 19.3)
Estimate the current bed capacity utilization of your department	49.6 (± 24.3)
Estimate the current reduction in operating room capacity in your department	54.2 (± 19.1)
Estimate the current operating room capacity utilization of your department	53.2 (± 27.9)
Estimate the current reduction in the outpatient clinics capacity of your department	69.3 (± 23.2)
Estimate the current capacity utilization of the consulting hours of your department	41.1 (± 30.8)
Estimate the percentage of physician staff to be redistributed to other departments	13.1 (± 16.0)
Estimate the proportion of medical staff working "reduced hours" or "shift work"	11.5 (± 23.4)
Estimate the percentage of medical staff who have been infected with SARS-CoV-2	2.2 (± 7.6)
Estimate the loss of your 2020 sales targets	28.2 (± 12.9)
Estimate the loss of your targets for the case mix points	27.3 (± 12.5)
Estimate the loss of your Case Mix Index targets	24.4 (± 13.2)

Concerning their employees, 28.4% (n = 42/148) replied that not enough protective gear was available. 7.4% (n = 11/148) of the emergency operations had to be postponed or rescheduled (Table 3). It was predominantly assumed that the number of elective surgeries will increase again after the pandemic. 43.9% (n = 65/148) noticed a decrease in the number of emergency surgeries, 41.2% (n = 61/148) agreed that it remained the same and 14.9% (n = 22/148) stated increased numbers of emergency surgeries. The number of surgical emergencies in the emergency room had decreased for 63.5% (n = 94/148) of those surveyed and increased for 6.1% (n = 9/148); 27.7% (n = 41/148) said they had treated an equal number of emergencies. Furthermore, there was a clear effect on the treatment of oncological patients. 54.1% (n = 80/148) of the respondents stated that they treated fewer oncological patients in their outpatient clinics. The number of patients dropped by 41.9 ± 24.5%, while 30.4% (n = 45/148) reported no change in the number of consultations. An increase was stated only by 12 respondents (8.1%), 11 surgeons (7.4%) did not specify this (Table 4).

**Table 3:** Restructurings due to COVID-19 pandemic (Part 2)

Questions	Answers			
	Yes n (%)	No n (%)	Unknown n (%)	Not specified n (%)
Has your clinic management ordered overtime to be reduced?	114 (77.0)	32 (21.6)	1 (0.7)	1 (0.7)
Has vacation been ordered by your clinic management?	31 (20.9)	116 (78.4)	1 (0.7)	0 (0.0)
Has it been possible to provide adequate protective equipment for your staff?	103 (69.6)	42 (28.4)	3 (2.0)	0 (0.0)
Did emergency operations have to be postponed or rescheduled due to sickness absence?	11 (7.4)	136 (91.9)	0 (0.0)	1 (0.7)
Would you estimate at this point in time that failure to meet the targets would have financial consequences for your department?	67 (45.3)	58 (39.2)	23 (15.5)	0 (0.0)
Would you currently estimate that failure to meet the targets would have personnel consequences for your department?	45 (30.4)	74 (50.0)	28 (18.9)	1 (0.7)
Has bed capacity been reduced in other surgical departments?	137 (92.6)	7 (4.7)	3 (2.0)	1 (0.7)
Has the operating room capacity also been reduced in other surgical departments?	142 (95.9)	4 (2.7)	2 (1.4)	0 (0.0)

**Table 4:** Impact of the COVID-19 pandemic on current case numbers

Question	Answer	n (%)	Change in % Mean ± single standard deviation
The number of emergency operations has…	Increased	22 (14.9)	22.0 ± 13.1
	Remained the same	61 (41.2)	–
	Decreased	65 (43.9)	29.5 ± 18.1
	Not specified	0 (0.0)	–
The number of surgical emergencies in the emergency room has…	Increased	9 (6.1)	22.2 ± 6.7
	Remained the same	41 (27.7)	–
	Decreased	94 (63.5)	36.3 ± 19.3
	Not specified	4 (2.7)	–
The number of oncological patients in the consultation hours has…	Increased	12 (8.1)	36.9 ± 28.6
	Remained the same	45 (30.4)	–
	Decreased	80 (54.1)	41.9 ± 24.5
	Not specified	11 (7.4)	–
Do you have the impression that patients cancel elective surgeries out of fear of a COVID-19 infection?	Yes	144 (97.3)	–
	No	3 (2.0)	–
	Unknown	1 (0.7)	–

### Outlook for the time after the early phase of the COVID-19 pandemic

**Fig. 2:**
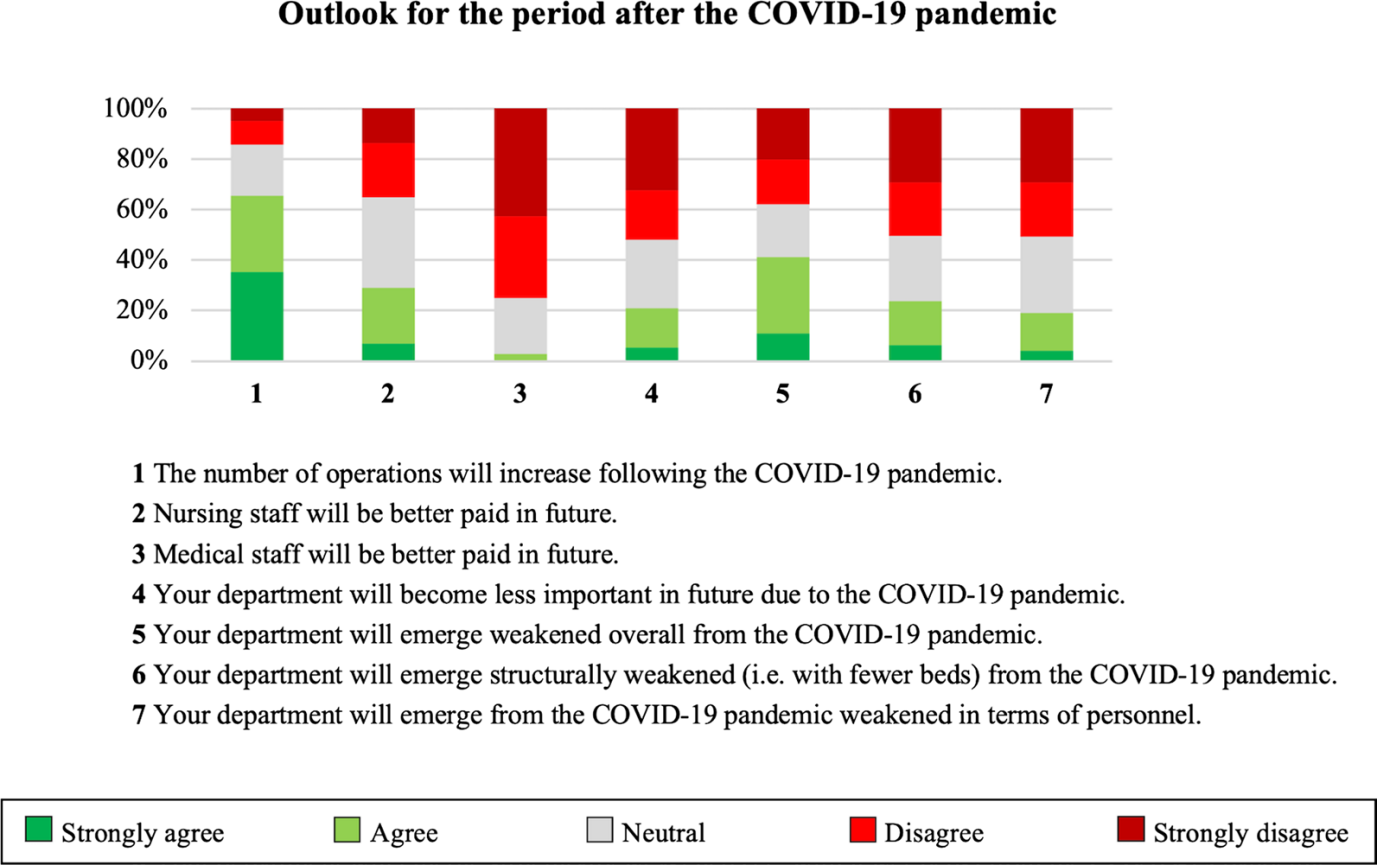
Results of questionnaire section 8: Outlook for the period after the COVID-19 pandemic, 8 questions

The study participants were asked about the effects of the pandemic on the payment of nurses and physicians. Approximately 30% of the chief surgeons assumed that nursing staff in particular will be better paid in the future. In contrast, only 2.7% (n = 4/148) of the respondents agreed with the statement that physicians will be paid better in the future (no one strongly agreed). Only 5.4% (n = 8/148) fully agreed and 15.5% (n = 23/148) agreed that the surgical departments will emerge weakened after the COVID-19 pandemic, whereas a total of 52.0% (n = 77/148) disagreed with this statement (Fig. 2). 50.7% (n = 75/148) and 50.4% (n = 74/148) of the study participants, respectively, assumed that the number of medical personnel and beds would not change, while 19.0% (n = 28/148) and 23.8% (n = 35/148) agreed that their department would be weaker in terms of staff and beds after the pandemic. At the end of the survey, the participants could give their feedback on the questionnaire and the situation in a free text answer. A total of 28 surgeons responded. In summary, the complete discontinuation of elective surgeries was predominantly perceived negatively and unsettled many surgeons.

## Discussion

This cross-sectional study provides data and assessments on the impact of the COVID-19 pandemic on the work of non-academic departments of surgery in Germany during the first lockdown and suspension of all elective surgeries. Altogether 152 members of the KLK answered the survey, 148 answers were included in the analysis.

The measures taken by politicians to contain the COVID-19 pandemic were mostly positively received by the heads of the surgical departments questioned in this survey. This is an important fact, considering that Germany introduced decentralized testing and initiated an early shutdown to flatten the epidemic curve. As a possible consequence, the death rate remained much lower than in France, Italy or Spain [13]. For future pandemic waves, this result could indicate that far-reaching and consistent political measures are well accepted by surgeons. Anyhow, the national suspension of the surgical programme was only well accepted by 47.9% of the participants, 16.2% were neutral and 35.8% disagreed. Another complete shut down of elective surgeries must be avoided. To address this point, we propose that, depending on the number of new COVID-19 infections in a particular region, only beds in hospitals in that region (and not at a national level) should be reserved and critical care capacity expanded. In addition, if necessary, another approach could be to restructure hospitals to treat only COVID-19 patients and keep other health care facilities COVID-19-free to maintain normal medical care.

The information policy of the health authories was as well largely perceived as positive, but the support of the hospital staff in general and the surgical departments with their special role in particular was judged insufficient by a majority of the responders. The cooperation between general and abdominal surgeons and their hospital administration was controversial, too. Communication was rated largely satisfactory, but in 71.7%, for example, no financial compensation was promised for the redistribution of staff, which can lead to a loss of confidence in the hospital management during further pandemic waves. Only one third of those interviewed considered their hospital management supportive. In comparison, the survey shows that the specialist disciplines worked well together indicating great trust and mutual support. Approximately one third of all respondents confirmed that initially not enough protective equipment was available at their hospitals and departments. This is not a singular problem, but rather that many healthcare facilities around the world initially lacked essential equipment such as disinfectants and personal protective equipment [14]. The shortage threatens the life of health care professionals in Germany and throughout the world. As a consequence, a sufficient storage for future pandemics have to be built up and made available more quickly. In addition, the supply of face masks and isolation material should be optimised; the Center for Disease Control and Prevention (CDC) of the US, for example, makes recommendations in this regard [15].

As mentioned before, in mid-March the German Government advised all hospitals and surgeons to postpone all scheduled admissions and operations if not absolutely necessary [16]. To mitigate financial losses for the surgical disciplines, the "Hospital Relief Act COVID19" was passed, which refunds each reserved intensive care bed [17]. Since May the surgical programme has been resumed step by step [18]. Overall, this phase led to an estimated average reduction in bed capacity of 50.8 ± 19.3% and operating room capacity of 54.2 ± 19.1%. The utilization of the reduced operating room capacity was only 53.2 ± 27.9% on average. The reduction in bed and surgical capacity led most respondents to estimate a loss of revenue of 28.2 ± 12.9% for whole 2020. In view of the incisive restrictions, it seems difficult to compensate for the economic losses from own resources. This is aggravated by the fact that the population is obviously uncertain about COVID-19, as only half of the available operating room capacity was needed. In addition, at the time of the survey, most hospitals had also reduced their outpatient clinics capacity, which might prolong the period of reduced operation rates. Once measures are scaled back, political support will be urgently needed to compensate for financial losses. Furthermore, the public must be made aware that hospital treatment is safe and that measures have been taken to avoid increasing the risk of infection with COVID-19.

The significant reduction in operative capacity and the redeployment of staff members in most facilities lead to concerns that urgent or emergency operations could not have been carried out. However, the survey showed that 91.9% of the participants were able to perform emergency operations without any restrictions or delay. Interestingly, almost half of the respondents (43.9%) stated that the number of emergency operations had dropped on average by one third. The same number of respondents reported no change in the number of emergency operations. The statement about surgical emergencies in the emergency room was even clearer. Almost two thirds (63.5%) reported a decreased number of admissions. The recorded numbers as well as previously reported data indicate, that patients might avoid attendance to the hospital even in urgent cases because they fear a COVID-19 infection [19]. Thus, initially elective operations will become emergencies in the future, which might lead to worse surgical care and postoperative outcome for the population in general and higher financial costs for the health care system.

The situation is similar with regard to the care of oncological patients. Whereas the interdisciplinary communication in tumour boards was widely unaffected, more than half of the respondents (54.1%) saw fewer patients in their outpatient clinic for both first consultation or follow-up care. This may lead to a deterioration in the early treatment or the detection of recurrences and thus impair the quality of oncological treatment in surgery. Further, the vast majority of the participants (97.3%) had to postpone or cancel a great number of elective surgeries and consultation appointments. The number of postponed treatments of patients in general and oncological patients in particular is obviously considerable. Nonetheless, exact numbers have to be further investigated, since it may have an impact on patient survival in oncological and non-oncological diseases.

Postponement of surgery during the pandemic is necessary not only to reserve beds for COVID-19 patients, but also because patients undergoing surgery are a vulnerable group at risk of hospital exposure to SARS-CoV-2. A recently published study showed that postoperative pulmonary complications occur in half of patients with perioperative SARS-CoV-2 infection and are associated with high mortality [20]. Therefore, the authors propose to consider postponing non-urgent interventions, especially in multimorbid patients, and to promote non-operative treatment. Additionally, the implementation of triage plans to prioritize operations appears essential. For instance, Ke et al. published strategies for the management of gastrointestinal surgery during COVID-19 [21]. Another research group from the UK published a broad overview of surgical practice during the pandemic [22, 23]. However, the elective surgical programme will be fully resumed at some point, and the postponed operations will need to be performed additionally. There is concern that the actual capacities together with the increased demand may not suffice timely surgical care for all patients in need. In regards to a recent study that estimates the total number of operations cancelled due to COVID-19 at almost 30 million, it is imperative to implement procedures allocating operating room capacity based on medical priority [24].

The present cross-sectional study is of course also subject to limitations. On the one hand, the collected data are based on subjective assessments, on the other hand, the survey was conducted in an early phase of the COVID-19 pandemic. Therefore, the results are to be considered preliminary and the future development and final impact has to be evaluated in additional investigations. Furthermore, the limited number of participants must be considered a limitation of the study.

## Conclusions

This survey presents the manifold impact of the COVID-19 pandemic on non-university surgical departments throughout Germany. It provides an overview of the challenges for the surgical care of patients in times of a pandemic. During the survey period, surgical departments in Germany faced the early stage of the COVID-19 pandemic and the gradual resumption of planned operations was carried out. It remains to determine whether the reduction in surgical capacity and the discontinuation of elective operations will lead to a reduction in the quality of surgical care in Germany. Furthermore, a possible deterioration in care and outcomes for oncological patients have to be analysed timely. Therefore, follow-up surveys are planned to provide further insights into this topic in the future with special emphasis on oncological results. In addition, the coordination of European and national measures for the care of our patients in the time of an ongoing pandemic should be based on the experience of clinicians and nursing staff in close cooperation with the politics. Our findings may help to adapt containment and restructuring strategies with regard to care reality in hospitals and to find comprehensive solutions with respect to possible future pandemic waves.

## Supplementary information

**Additional file 1: Table S1.** Membership structure of *Konvent der leitenden Krankenhauschirurgen* (Convention of leading Hospital Surgeons).

**Additional file 2:** Survey.

**Additional file 3: Figure S1.** Interdisciplinary tumour board.

## Data Availability

The datasets used and/or analysed during the current study are available from the corresponding author on reasonable request.

## Abbreviations

WHO: World Health Organization
KLK: Convention of leading hospital surgeons
CDC: Center for disease control and prevention
