# Supplementary material for: The COVID-19 pandemic: impact on surgical departments of non-university hospitals
Source: BMC Surg. 2020 Dec 3;20:313. doi: 10.1186/s12893-020-00970-x (PMC7711305; doi:10.1186/s12893-020-00970-x)
Supplement: Supplementary file 1 — Additional file 1: Table S1. Membership structure of Konvent der leitenden Krankenhauschirurgen (Convention of leading Hospital Surgeons). [file 12893_2020_970_MOESM1_ESM.docx]

**Table S1: Membership structure of *Konvent der leitenden Krankenhauschirurgen* (Convention of leading Hospital Surgeons)**

| **Specialty** | **n** |
| --- | --- |
| General, Abdominal and Thoracic Surgery | 409 |
| Trauma Surgery/Orthopedics | 49 |
| Plastic/Reconstructive Surgery | 4 |
| Paediatric Surgery | 2 |
| Heart and Thoracic Surgery | 12 |
| Neurosurgery | 2 |
| Oral and Maxillofacial Surgery | 1 |
| Miscellaneous | 2 |
| Not specified | 328 |
