# Supplementary material for: The COVID-19 pandemic: impact on surgical departments of non-university hospitals
Source: BMC Surg. 2020 Dec 3;20:313. doi: 10.1186/s12893-020-00970-x (PMC7711305; doi:10.1186/s12893-020-00970-x)
Supplement: Supplementary file 2 — Additional file 2: Survey. [file 12893_2020_970_MOESM2_ESM.docx]

**The COVID-19 pandemic: impact on surgical departments of non-university hospitals**

***Survey***

Stöß et al.

**General characteristics**

**1. Your age**

> 50 years ☐

30-50 years ☐

< 30 years ☐

Not specified ☐

**2. Sex**

Male ☐

Female ☐

Non-binary ☐

Not specified ☐

**4. Profession (obligatory)**

Head of department ☐

Senior attending ☐

Consultant ☐

**5. Supply level of the hospital**

Maximum care hospital ☐

Specialized hospital ☐

General hospital ☐

**6. Hospital type**

Private carrier ☐

Public ☐

Non-profit carrier ☐

**7. Hospital size (number of beds)**

< 100 ☐

101-200 ☐

201-500 ☐

501-1000 ☐

> 1000 ☐

Not specified ☐

**8. Intensive care unit capacity (number of beds)**

< 10 ☐

11-20 ☐

21-50 ☐

51-100 ☐

> 100 ☐

Not specified ☐

**9. Please enter the number of operations performed annually by your surgical department.**

**Politics**

**10. Information on the development of the COVID-19 pandemic was sufficiently disseminated**

(1) Strongly agree (2) (3) (4) (5) Strongly disagree

**11. The measures taken to contain the COVID-19 pandemic were adequate.**

(1) Strongly agree (2) (3) (4) (5) Strongly disagree

**12. The suspension of all elective operations was an adequate measure.**

(1) Strongly agree (2) (3) (4) (5) Strongly disagree

**13. More commitments for financial support or coverage would have been desirable.**

(1) Strongly agree (2) (3) (4) (5) Strongly disagree

**14. Despite the pandemic, the work of our department has been perceived as important.**

(1) Strongly agree (2) (3) (4) (5) Strongly disagree

**Health authorities**

**15. The information policy of the health authorities was adequate.**

(1) Strongly agree (2) (3) (4) (5) Strongly disagree

**16. The health authorities have taken adequate measures to protect hospital staff.**

(1) Strongly agree (2) (3) (4) (5) Strongly disagree

**17. The federal health authorities were perceived as supporters of our department.**

(1) Strongly agree (2) (3) (4) (5) Strongly disagree

**18. The state health authorities were perceived as supporters of our department.**

(1) Strongly agree (2) (3) (4) (5) Strongly disagree

**Hospital administration**

**19. Communication with your hospital administration was adequate.**

(1) Strongly agree (2) (3) (4) (5) Strongly disagree

**20. Your hospital administration has clearly communicated that financial targets in this situation are subordinate.**

(1) Strongly agree (2) (3) (4) (5) Strongly disagree

**21. Your hospital administration has intervened with the personnel allocation.**

(1) Strongly agree (2) (3) (4) (5) Strongly disagree

**22. There was financial compensation for the reallocation of your staff to other departments.**

(1) Strongly agree (2) (3) (4) (5) Strongly disagree

**23. Training courses on the handling of COVID-19 patients were offered to the staff.**

(1) Strongly agree (2) (3) (4) (5) Strongly disagree

**24. Your hospital administration has ensured adequate protection for the staff.**

(1) Strongly agree (2) (3) (4) (5) Strongly disagree

**25. Your hospital administration has been appointed as a supporter of the general and abdominal surgery departments.**

(1) Strongly agree (2) (3) (4) (5) Strongly disagree

**26. Your hospital administration assures that surgical wards reserved for COVID-19 patients will be released for surgery again after the pandemic.**

(1) Strongly agree (2) (3) (4) (5) Strongly disagree

**Cooperation with other specialties during COVID-19 pandemic**

**27. Communication with the anaesthetists/intensive care physicians was adequate.**

(1) Strongly agree (2) (3) (4) (5) Strongly disagree

**28. Communication with infectiologists/virologists was adequate.**

(1) Strongly agree (2) (3) (4) (5) Strongly disagree

**29. Communication with gastroenterologists/oncologists was adequate.**

(1) Strongly agree (2) (3) (4) (5) Strongly disagree

**30. A regular interdisciplinary exchange of measures to be coordinated with regard to of the distribution of COVID-19 patients took place.**

(1) Strongly agree (2) (3) (4) (5) Strongly disagree

**31. The interdisciplinary tumor board continues to take place.**

Yes, as before. ☐

Yes, but with less staff. ☐

Yes, but only via video/phone conference. ☐

No, the tumor board is currently paused/reduced. ☐

Not specified ☐

**Restructuring / pandemic consequences**

**32. Estimate the current reduction in bed capacity in your department as a result of the pandemic**. *(If the bed capacity of your clinic has increased or remained the same, please enter a "0" here)*

**33. Estimate the current bed capacity utilization of your department as a result of the pandemic.**

**34. Estimate the current reduction in operating room capacity in your department as a result of the pandemic.** *(If the operating room capacity of your clinic has increased or remained the same, please enter a "0" here)*

**35. Estimate the current operating room capacity utilization of your department as a result of the pandemic.**

**36. Estimate the current reduction in the consulting room capacity of your department as a result of the pandemic.** *(If the consultation hours of your clinic have increased or remained the same, please enter a "0" here)*

**37. Estimate the current capacity utilization of the consulting hours of your department as a result of the pandemic.**

**38. Has bed capacity been reduced in other operational departments?**

Yes ☐

No ☐

Unkown ☐

**39. Has the operating room capacity also been reduced in other surgical departments?**

Yes ☐

No ☐

Unkown ☐

**40. Estimate the percentage of physician staff to be redistributed from your department to other departments.**

**41. To which departments were the employees reallocated (e.g. gastroenterology, intensive care unit etc.)?**

Free-text

**42. Estimate the proportion of medical staff in your department working "reduced hours" or "shift work".**

**43. Estimate the percentage of medical staff in your department who have been infected with COVID-19.**

**44. Has your clinic management ordered overtime to be reduced?**

Yes ☐

No ☐

Unkown ☐

**45. Has vacation been ordered by your clinic management?**

Yes ☐

No ☐

Unkown ☐

**46. Has it been possible to provide adequate protective equipment for your staff?**

Yes ☐

No ☐

Unkown ☐

**47. Did emergency operations have to be postponed or rescheduled due to sickness absence?**

Yes ☐

No ☐

Unkown ☐

**48. Would you estimate at this point in time that failure to meet the targets would have financial consequences for your department?**

Yes ☐

No ☐

Unkown ☐

**49. Would you currently estimate that failure to meet the targets would have personnel consequences for your department?**

Yes ☐

No ☐

Unkown ☐

**50. Estimate the loss of your 2020 sales targets.** *(Should the revenue at your clinic increase or remain the same, please enter a "0" here)*

**51. Estimate the loss of your targets for the case mix points**. *(Should the Case Mix points at your clinic increase or remain the same, please enter a "0" here)*

**52. Estimate the loss of your Case Mix Index targets.** *(Should the Case Mix Index at your clinic increase or remain the same, please enter a "0" here)*

**Impact of the COVID-19 pandemic on current case numbers**

**53. The number of emergency operations has increased (indicated in %).**

*(If the number of emergency operations has decreased or remained the same, please enter a "0" here)*

**54. The number of emergency operations has decreased (indicated in %)**

*(If the number of emergency operations has increased or remained the same, please enter a "0" here)*

**55. The number of surgical emergencies in the emergency room has increased indicated in %).**

*(If the number of surgical emergencies in the emergency department has decreased or remained the same, please enter a "0" here).*

**56. The number of surgical emergencies in the emergency room has decreased (indicated in %).**

*(If the number of surgical emergencies in the emergency department has increased or remained the same, please enter a "0" here).*

**57. The number of oncological patients in consultation hours has increased (indicated in %).**

*(If the number of oncological patients in the consultation hour has decreased or remained the same, please enter a "0" here)*

**58. The number of oncological patients in consultation hours has decreased (indicated in %).**

*(If the number of oncological patients during consultation hours has increased or remained the same, please enter a "0" here).*

**59. Do you have the impression that patients are worried about COVID-19 infection cancel elective surgery?**

Yes ☐

No ☐

Unkown ☐

**Outlook for the period after the COVID-19 pandemic**

**60. The number of operations will increase following the COVID-19 pandemic.**

(1) Strongly agree (2) (3) (4) (5) Strongly disagree

**61. Nursing staff will be better paid in future.**

(1) Strongly agree (2) (3) (4) (5) Strongly disagree

**62. Medical staff will be better paid in future.**

(1) Strongly agree (2) (3) (4) (5) Strongly disagree

**63. Your department will become less important in future due to the COVID-19 pandemic.**

(1) Strongly agree (2) (3) (4) (5) Strongly disagree

**64** **Your department will emerge weakened overall from the COVID-19 pandemic.**

(1) Strongly agree (2) (3) (4) (5) Strongly disagree

**65. Your department will emerge structurally weakened (i.e. with fewer beds) from the COVID-19 pandemic.**

(1) Strongly agree (2) (3) (4) (5) Strongly disagree

**66. Your department will emerge from the COVID-19 pandemic weakened in terms of personnel.**

(1) Strongly agree (2) (3) (4) (5) Strongly disagree

**67. Feel free to leave a comment or feedback.**

Free text
