# Supplementary figures and images for: The COVID-19 pandemic: impact on surgical departments of non-university hospitals
Source: BMC Surg. 2020 Dec 3;20:313. doi: 10.1186/s12893-020-00970-x (PMC7711305; doi:10.1186/s12893-020-00970-x)

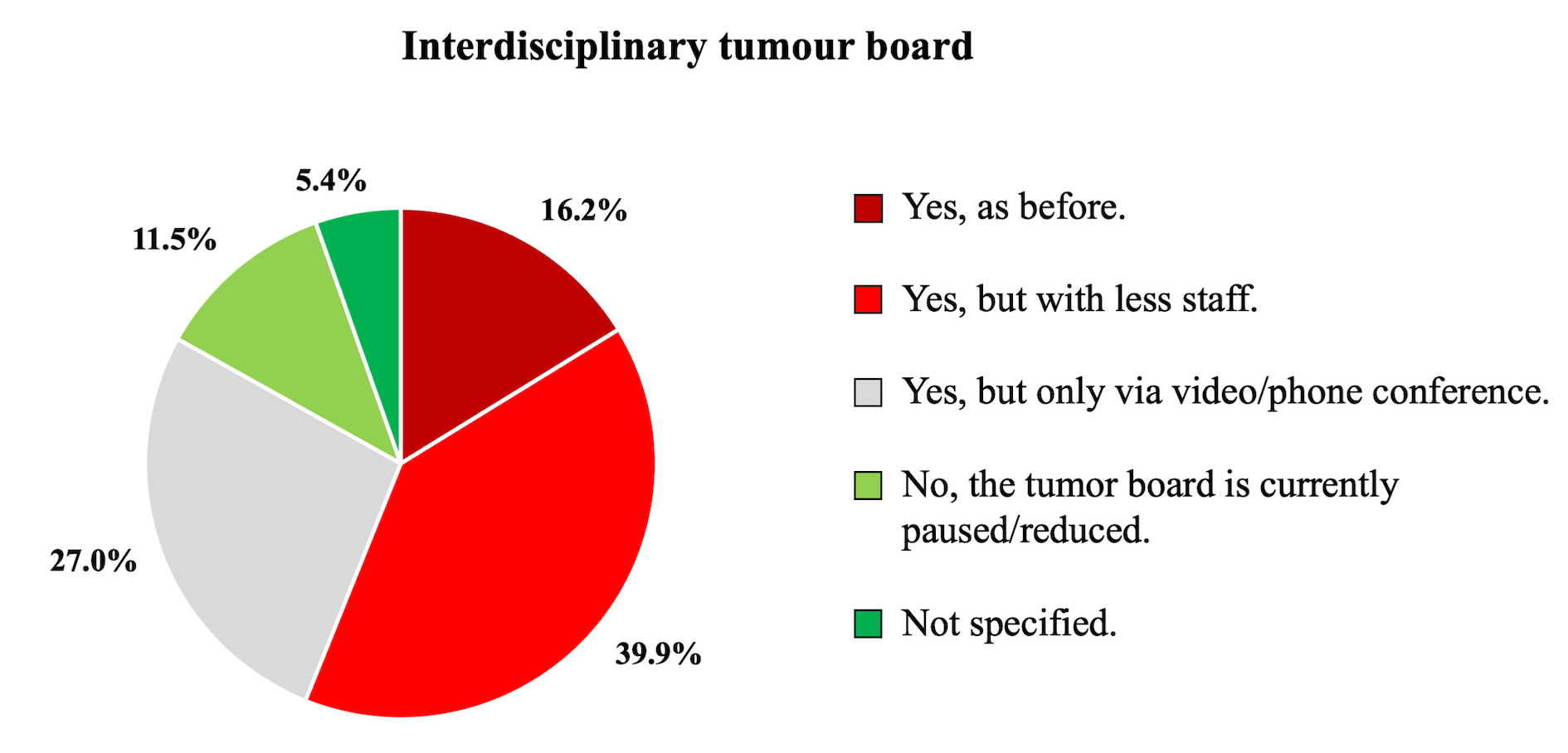

Supplement: Supplementary file 3 — Additional file 3: Figure S1. Interdisciplinary tumour board. [file 12893_2020_970_MOESM3_ESM.tiff]
